# A New Error Model and Compensation Strategy of Angle Encoder in Torsional Characteristic Measurement System

**DOI:** 10.3390/s19173772

**Published:** 2019-08-30

**Authors:** Bingkui Chen, Changyan Peng, Jian Huang

**Affiliations:** State Key Laboratory of Mechanical Transmission, Chongqing University, Chongqing 400044, China

**Keywords:** angle encoder, torsional characteristic measurement, geometric error, error compensation, multibody system theory

## Abstract

For systems of measurement, geometric errors such as manufacturing and assembly errors could have a significant impact on the accuracy of the angle encoders of the system. In this study, an error model of angular measurement with geometric errors of a torsional characteristic measurement system was developed based on multibody system theory, the aim of which was to reveal the impact of geometric errors on angular measurement and to compensate the measurement error. According to the principle of spatial error transfer, the decomposition of geometric errors is illustrated and the error matrix of geometric errors is constructed by the Denavit–Hartenberg (DH) method. Subsequently, an error compensation function can be obtained and the impact of geometric error on angular measurement is discussed. Finally, we demonstrate by the experimental results of an ultra-autocollimator that the proposed error compensation method reduced the angular measurement error from 3.7% to 0.7%, which shows that the proposed error model can effectively predict the angular measurement error. In addition, it can be seen from the measurement results of the RV reducer that the error of the torsional characteristic measurement system decreased significantly.

## 1. Introduction

The torsional characteristic measurement system (TCMS) can be used to measure the hysteresis curve, torsional stiffness, and backlash of precision reducers for robots, such as an RV reducer and harmonic drive [1]. In addition, the performance and application range of the TCMS are directly determined by the measurement accuracy of the angle. With the improvement of the efficiency and accuracy of the measurement system, the circular grating angle encoder (CGAE) is more commonly used in the field of torsional characteristic measurement because of its good stability, convenient installation, high precision, and fast speed [2,3]. However, the geometric errors of the system, such as part manufacturing tolerances and assembly error, could potentially affect the measurement accuracy of the angle encoder [4]. Therefore, it is desirable to investigate the error feature and compensation strategy of an angle encoder to enhance the measurement accuracy of the TCMS.

In recent studies, the TCMS of precision reducers mainly depends on high-precision angle-measuring equipment in order to ensure the system measurement accuracy. There have been many different torsion angle measurement methods proposed in the literature. For example, Fan and Zhang proposed a hysteretic curve measurement system based on a photoelectric encoder, and its resolution to the angle of rotation was 0.1 arcmin [5]. Michael and Torsten constructed a set of torsional characteristic testing systems using an angle encoder that could be used to measure the torsion angle of discrete points for the harmonic drive. Moreover, the measurement data were compared with that from a theoretical model [6]. Zheng and Xi developed a dynamic characteristics test rig for an RV reducer, where the rail mounting was used to reduce the influence of installation error on the angle encoder [7]. Sun and Han used a laser instrument directly mounted at the output of the reducer to measure the hysteresis curve of a CBR reducer [8]. The measurement error of the torsion angle has a greater impact on the evaluation of the torsional characteristics than the measurement and evaluation of the rotation angle. For example, when evaluating the torsional stiffness of an RV reducer, an angular measurement error of 20 arcsec would result in a stiffness calculation error of approximately 10%. 

The study of angle sensor measurement error correction mainly focuses on two directions. The first direction is based on multiple sensors or multireading heads for error compensation. Ai and Chu proposed a method to detect the magnitude and direction of eccentricity for a circular grating angle encoder by using two read heads at 180° [9]. Lou and Hao proposed a multiread head measurement method to automatically compensate for circular grating deformation, manufacturing defects, and mounting errors. Substantially, it eliminated most of the error of the circular grating angle sensor [10]. Tsukasa et al. developed an automatic calibration system that automatically eliminated the eccentricity error of the rotating shaft using two angle encoders, and the compensation function was obtained by the equal division averaged (EDA) method [11]. However, multiple reading methods require higher mounting accuracy and more heads or sensors must be used, which adds significant cost. Therefore, many other studies have sought to improve the accuracy of circular grating angle encoders without using more read heads. In particular, Guo and Li analyzed the influence of eccentricity on the measurement accuracy of the angle sensor and used the quantitative analysis method to derive the measurement error compensation formula caused by the eccentricity [12]. Gao and Wang proposed a CGAE error compensation model using calibration data and the FFT analysis method. The parameters of the compensation model were determined by the particle swarm optimization algorithm [13]. A mathematical model was proposed by Su and Qiu to reveal the effect of the magnitude and direction of the eccentricity on the angular measurement error [14]. Mi and Gao validated the law of the sinusoidal variation of angle measurement error caused by the installation of eccentricity and angular variation and corrected the error according to the least-squares method (LSM) [15]. These studies are mainly based on calibration data to analyze the relationship between eccentricity error and measurement error and then establish a compensation strategy. However, these methods are very time consuming when calibrating the system for each measurement and only consider the effect of the eccentricity error. Therefore, the measurement error prediction and model analysis of the angle sensor considering the geometric error are of great significance for improving the measurement accuracy and the application of a sensor in different engineering measurement situations.

Based on the above discussion, this paper proposes a new circular grating angle encoder measurement error compensation model, which takes into account the geometric errors of the measurement unit. Compared with traditional error compensation methods, the proposed compensation strategy is more comprehensive and suitable for high-precision angle encoder applications. In Section 2, the geometric errors affecting the measurement accuracy of the TCMS angle encoder are analyzed, and the measurement model of the angle encoder under geometric error is proposed. Section 3 uses the Denavit–Hartenberg (DH) method to establish a mathematical model of the measurement error and derives the parameter function of the measurement error. In Section 4, the effects of these errors on the accuracy of the angular encoder are discussed and calibration experiments are performed to validate the proposed error compensation model. 

## 2. Measurement Model of Angle Encoder on TCMS

### 2.1. Measurement System

**Figure 1:**
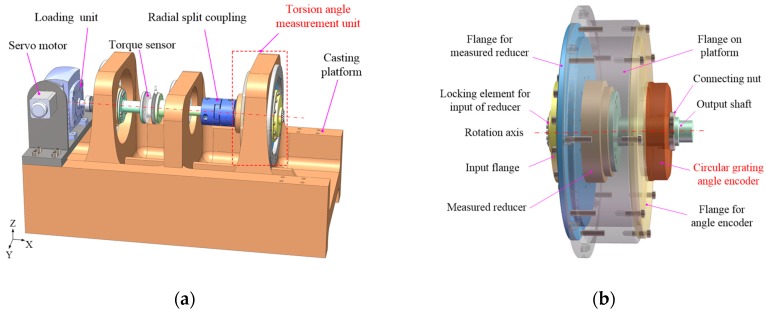
(**a**) The structure diagram of the torsional characteristic measurement system (TCMS). (**b**) The structure diagram of the torsion angle measurement unit.

In this study, the structure of a TCMS for a precision reducer was developed, as shown in Figure 1a. The main structure of the system consisted of a casting platform, a torsion angle measurement unit, a torque sensor, a loading unit, a servomotor, and a control system for motion control and data acquisition. In order to meet the requirements of the multimodel precision reducer and improve measurement efficiency, a circular grating angle encoder (Heidenhain RON786-18000 with a radius of rotation of 30 mm, a resolution of 0.7 arcsec after subdivision, and a system accuracy of ±2 arcsec) was used in this system, and a simplified measurement process was designed. Firstly, the measured reducer, the output shaft, and the flange for the measured reducer were assembled into a corresponding relationship. Then, the above components were mounted on a flange fixed on the casting platform, and the output shaft and angle encoder were connected by a nut, as shown in Figure 1b.

### 2.2. Coordinate System and Geometric Source Errors

**Figure 2:**
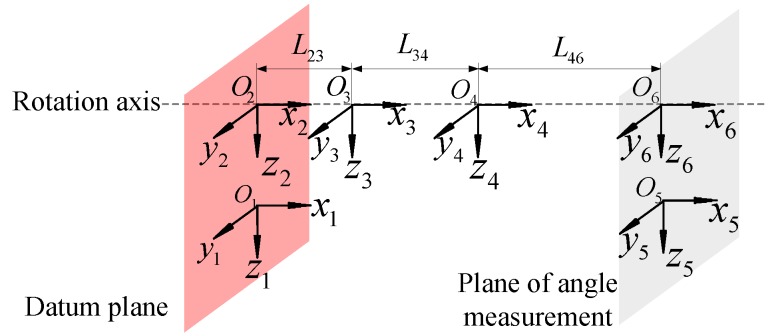
Schematic for corresponding coordinate system of the torsion angle measurement unit.

In order to quantitatively analyze geometric errors, we used the corresponding coordinate system of the torsion angle measurement unit, as shown in Figure 2, which is based on the mounting relationship of the parts. Each coordinate of the part was set as follows:

- Coordinate 1: platform coordinate *S1*, $O_{1}-x_{1}y_{1}z_{1}$
- Coordinate 2: flange for measured reducer *S2*, $O_{2}-x_{2}y_{2}z_{2}$
- Coordinate 3: measured reducer *S3*, $O_{3}-x_{3}y_{3}z_{3}$
- Coordinate 4: output shaft *S4*, $O_{4}-x_{4}y_{4}z_{4}$
- Coordinate 5: flange for angle encoder *S5*, $O_{5}-x_{5}y_{5}z_{5}$
- Coordinate 6: platform coordinate *S6*, $O_{6}-x_{6}y_{6}z_{6}$

According to the analysis proposed in [16,17], the geometric error of the measurement system refers to the manufacturing error of each component and the assembly error between the components. Figure 1b and Figure 2 show that the errors caused by the installation process in the *x*-axis and *oyz* plane are sensitive sources of measurement error. Here, for a component, we define Δl as the length dimension error along the *x*-axis, Δτ as the perpendicularity error,Δd as the diameter error, and Δc as the coaxial error. For the adjacent components after installation, the relative position error along the *x*-axis, the eccentricity error of the *x*-axis, and the tilt errors along the mounting surface are defined as δr, δe, and δt, respectively. The relationship between the manufacturing errors and the assembly errors for adjacent parts can be expressed as

(1) $$\{\begin{array}{c} \delta r_{ij}=\Delta l_{i}+\Delta l_{j}\\ \delta e_{ij}=\left|\Delta d_{i}-\Delta d_{j}\right|+\Delta c_{i}\\ \delta t_{ij}=\Delta \tau_{i}+\Delta \tau_{j} \end{array}$$

where i and j are the component number.

### 2.3. Measurement Model of Angle Encoder under Geometric Errors 

**Figure 3:**
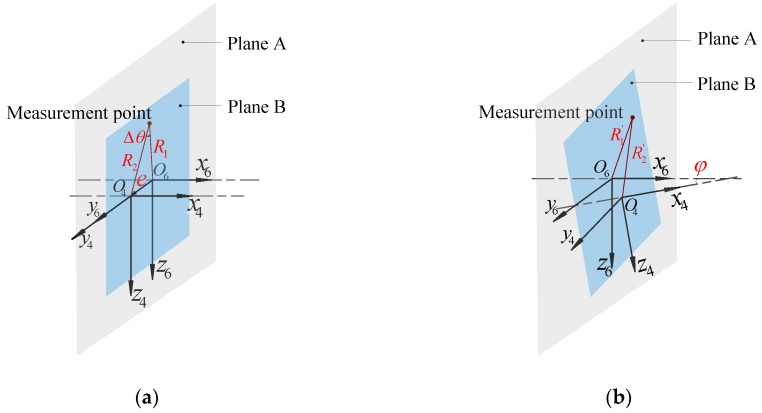
(**a**) Angle measurement relationship only considering eccentricity. (**b**) Angle measurement relationship considering geometric errors.

Based on the spatial coordinate system and angle measurement relationship established in the previous section, the angle measurement model was constructed under error, as shown in Figure 3. Under the impact of geometric errors, the position of the measuring point changes. The measurement plane in which the sensor is located is defined as plane A, and the plane of the measured point on the output shaft is defined as plane B. Ideally, the sensor and output shaft rotate about the *x*-axis. The traditional analysis method only considers the influence of the eccentricity error of the rotation axis. Moreover, the measurement model of the angle encoder under eccentricity error is shown in Figure 3a. The angular error can be expressed as [9]

(2) $$\{\begin{array}{c} \Delta \theta =\arctan\frac{e\cdot \sin\theta }{R_{1}-e\cos\theta }\\ \varphi =0 \end{array}$$

where $R_{1}$ is the rotation radius of the angle encoder, e is the eccentric error, θ is the measurement angle by the encoder, and φ is the stagger angle of the output shaft axis and the rotation axis of the angle encoder.

However, under the influence of geometrical errors, the axis of rotation of the output shaft and the axis of rotation of the encoder produce staggered angles. The measurement model under geometric errors is shown in Figure 3b. Based on the basic principle of spatial meshing [18], the angular error can be expressed as a function related to $R_{1}^{\prime }$ and $R_{2}^{\prime }$:

(3) $$\{\begin{array}{c} \Delta \theta^{\prime }=f(R_{1}^{\prime },R_{2}^{\prime },\theta )\\ \varphi \neq 0 \end{array}$$

where $R_{1}^{\prime }$ is the rotation radius of the actual measurement point on the angle encoder, and $R_{2}^{\prime }$ is the radius of the actual measurement point relative to the axis on the output shaft section.

## 3. Mathematical Model of Error Compensation

In this section, based on the DH method, the relationship between the position of the measuring point and the manufacturing and assembly errors of each component is established, and then the error compensation model of the angle sensor is developed.

### 3.1. Error Matrix and Geometric Error Decomposition

Based on the basic principle of the multibody system error model [19], when there is a geometric error adjacent part coordinate system, it can be decomposed according to the sensitive direction of the error, which can be expressed as Δx, Δy, Δz, Δα, Δβ, and Δγ, respectively. It should be noted that Δx, Δy, and Δz are the translation errors along the *x*-axis, *y*-axis, and *z*-axis, and Δα, Δβ, and Δγ are the rotation errors around the axis, respectively. Therefore, according to the DH theory and the simplified method in [20], the general expression of the characteristic matrix of errors can be expressed as

(4) $$\Delta T_{ij}=\left[\begin{array}{cccc} 1 & -\Delta \gamma_{ij} & \Delta \beta_{ij} & \Delta x_{ij}\\ \Delta \gamma_{ij} & 1 & -\Delta \alpha_{ij} & \Delta y_{ij}\\ -\Delta \beta_{ij} & \Delta \alpha_{ij} & 1 & \Delta z_{ij}\\ 0 & 0 & 0 & 1 \end{array}\right]$$

where $\Delta T_{ij}$ (i and j are the component numbers) represents an error matrix between adjacent parts. 

It can be obtained that the error transmission direction is S1-S2-S3-S4 and S1-S5-S6 from the coordinate systems established in Section 2. Taking the S1 and S2 coordinate systems as an example, the geometric errors of the adjacent coordinate system are decomposed as follows:

(1) Determine the relationship between the manufacturing and assembly errors of adjacent components in S1 and S2 coordinate systems based on Equation (1), and the relationship can be expressed as
  (5) $$\{\begin{array}{c} \delta r_{12}=0\\ \delta e_{12}=|\Delta d_{1}-\Delta d_{2}|\\ \delta t_{12}=\Delta \tau_{1}+\Delta \tau_{2} \end{array}.$$
(2) Decompose the above assembly errors according to the translation and rotation errors in Equation (4). Simultaneously, the transformed feature matrix $T_{12}$ can be obtained based on the position relationship between coordinate systems. The feature matrix $T_{12}$ and error matrix $\Delta T_{12}$ of S2 relative to S1 can be expressed as
  (6) $$T_{12}=\left[\begin{array}{cccc} 1 & 0 & 0 & 0\\ 0 & 1 & 0 & 0\\ 0 & 0 & 1 & 0\\ 0 & 0 & 0 & 1 \end{array}\right],\Delta T_{12}=\left[\begin{array}{cccc} 1 & -\delta t_{12} & \delta t_{12} & 0\\ \delta t_{12} & 1 & 0 & \delta e_{12}\\ -\delta t_{12} & 0 & 1 & \delta e_{12}\\ 0 & 0 & 0 & 1 \end{array}\right].$$
(3) Repeat (1) and (2) for the other adjacent coordinate systems. The feature matrix $T_{ij}$ and the relationships between the manufacturing and assembly errors of other adjacent components are obtained as follows:
  (7) $$T_{23}=\left[\begin{array}{cccc} 1 & 0 & 0 & L_{23}\\ 0 & 1 & 0 & 0\\ 0 & 0 & 1 & 0\\ 0 & 0 & 0 & 1 \end{array}\right],T_{34}=\left[\begin{array}{cccc} 1 & 0 & 0 & L_{34}\\ 0 & 1 & 0 & 0\\ 0 & 0 & 1 & 0\\ 0 & 0 & 0 & 1 \end{array}\right],T_{15}=\left[\begin{array}{cccc} 1 & 0 & 0 & L_{15}\\ 0 & 1 & 0 & 0\\ 0 & 0 & 1 & 0\\ 0 & 0 & 0 & 1 \end{array}\right],T_{56}=\left[\begin{array}{cccc} 1 & 0 & 0 & 0\\ 0 & 1 & 0 & 0\\ 0 & 0 & 1 & 0\\ 0 & 0 & 0 & 1 \end{array}\right]$$
  (8) $$\{\begin{array}{c} \delta r_{23}=\Delta l_{2}+\Delta l_{3}\\ \delta e_{23}=|\Delta d_{2}-\Delta d_{3}|+\Delta c_{2}\\ \delta t_{23}=\Delta \tau_{2}+\Delta \tau_{3} \end{array},\{\begin{array}{c} \delta r_{34}=\Delta l_{3}+\Delta l_{4}\\ \delta e_{34}=|\Delta d_{4}-\Delta d_{3}|\\ \delta t_{34}=\Delta \tau_{3}+\Delta \tau_{4} \end{array},\{\begin{array}{c} \delta r_{15}=\Delta l_{5}\\ \delta e_{15}=|\Delta d_{1}-\Delta d_{5}|\\ \delta t_{15}=\Delta \tau_{1}+\Delta \tau_{5} \end{array},\{\begin{array}{c} \delta r_{56}=0\\ \delta e_{56}=|\Delta d_{5}-\Delta d_{6}|\\ \delta t_{56}=\Delta \tau_{5}+\Delta \tau_{6} \end{array}.$$

According to the above analysis, the errors between adjacent parts of the torsion angle measurement unit after decomposition are shown in Table 1.

### 3.2. The Transformation of the Measurement Point

Based on the principle of homogeneous coordinate transformation, the feature matrix $T_{ij}$ is used to describe the position coordinate transformation of the measurement point in each coordinate system. Assuming a measurement point $E_{t}$ in the output shaft coordinate system S4, a corresponding point $E_{m}$ can be obtained in the angle measurement coordinate system S6 after a series of coordinate transformations of the intermediate coordinate system. The transformation relationship of $E_{t}$ and $E_{m}$ can be expressed as

(9) $$\{\begin{array}{c} E_{m}=T_{64}\cdot E_{t}\\ \;T_{64}={\left(T_{15}\Delta T_{15}T_{56}\Delta T_{56}\right)}^{-1}T_{12}\Delta T_{12}T_{23}\Delta T_{23}T_{34}\Delta T_{34} \end{array}.$$

### 3.3. Derivation of Mathematical Model

**Figure 4:**
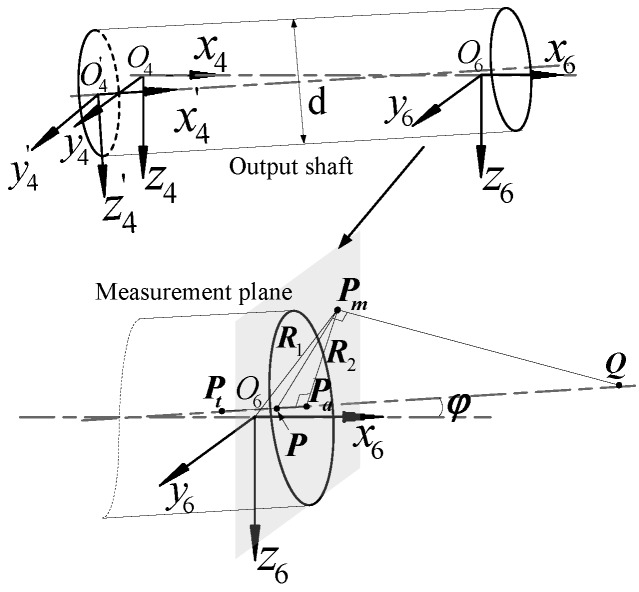
Geometric relationship of the measurement point under errors.

According to the measurement model proposed in Section 2, this subsection shows how we determined the relationship between the theoretical and actual measurement points based on the spatial geometry method [21]. Suppose *Pt* is the theoretical measurement point on the output shaft, *P* is the intersection of the actual rotation axis and the measurement plane, *Pa* is the actual measurement point on the output shaft, and the diameter of the output shaft is *d*. The coupling nut on the normal plane of the output shaft passes the point *Pa*, the intersection of the measurement plane is *Pm*, and *PaPm* = *R*_2_. The intersection point of the normal of the crossing point *Pm* and the actual output shaft on the measurement plane is *Q*, as shown in Figure 4.

According to the system structure parameters, the coordinates of point *Pt* in the S4 coordinate system are $\left(L_{47},\;0,\;0\right)$. In addition, two points (*P*_0_ and *P*_1_) on the *x*-axis are selected in the S4 coordinate system. The coordinates are converted according to Equation (9) to obtain corresponding points in the S6 coordinate system. The coordinate calculation formula can be expressed as

(10) $${P_{t}}^{\prime }=\left[\begin{array}{c} x_{t}\\ y_{t}\\ z_{t}\\ 1 \end{array}\right]{=T}_{64}\cdot \left[\begin{array}{c} L_{47}\\ 0\\ 0\\ 1 \end{array}\right]\;{P_{0}}^{\prime }=\left[\begin{array}{c} x_{0}\\ y_{0}\\ z_{0}\\ 1 \end{array}\right]=T_{64}\cdot \left[\begin{array}{c} 0\\ 0\\ 0\\ 1 \end{array}\right],\;{P_{1}}^{\prime }=\left[\begin{array}{c} x_{1}\\ y_{1}\\ z_{1}\\ 1 \end{array}\right]{=T}_{64}\cdot \left[\begin{array}{c} 1\\ 0\\ 0\\ 1 \end{array}\right].$$

To facilitate the derivation calculation, the transformation matrix T_64_ is represented by a parameter matrix as

(11) $$T_{64}=\left[\begin{array}{cccc} m_{11} & m_{12} & m_{13} & m_{14}\\ m_{21} & m_{22} & m_{23} & m_{24}\\ m_{31} & m_{32} & m_{33} & m_{34}\\ 0 & 0 & 0 & 1 \end{array}\right].$$

By combining Equations (10) and (11), the linear equation and unit direction vector along the actual axis of the output shaft can be expressed as

(12) $$\begin{array}{c} \frac{x-m_{14}}{m_{11}}=\frac{y-m_{24}}{m_{21}}=\frac{z-m_{34}}{m_{31}}\\ \vec{a}=\vec{{P_{0}}^{\prime }{P_{1}}^{\prime }}=(m_{11},\;m_{21},\;m_{31}) \end{array}.$$

In the S7 coordinate system, the unit normal vector of the measurement surface can be expressed as

(13) $$\vec{n}=(1,\;0,\;0).$$

By combining Equations (12) and (13), the angle between the actual axis of the output shaft and the axis of rotation of the angular encoder can be expressed as

(14) $$\varphi =\arccos(\frac{\vec{a}\cdot \vec{n}}{\left|\vec{a}\right|\cdot \left|\vec{n}\right|}).$$

Through Equations (10)–(14) and the positional relationship of each point in Figure 4, the lengths of $P_{m}O_{6}$ and $P_{a}P_{t}$ can be derived based on the spatial positional relationship:

(15) $$\left\{ \begin{array}{l} \begin{array}{c} P_{m}O_{6}=\sqrt{{\left(am_{11}+m_{14}\right)}^{2}+{\left(am_{21}+m_{24}\right)}^{2}}\\ a=\sqrt{\frac{R_{2}}{m_{11}\sin\varphi }}-\frac{m_{14}}{m_{11}} \end{array}\\ P_{a}P_{t}=\sqrt{R_{2}\tan\varphi }-\frac{m_{14}}{m_{11}}-L_{47} \end{array}\right..$$

Due to the influence of the geometric errors, the measurement area will change with the changes of the actual measurement point on the output shaft. To evaluate the measurement error accurately, we define $\Delta \theta_{S}$ as the angle measurement error caused by the changes of the measurement area. Based on the above equations, the mathematical function for the angle measurement error can be expressed as

(16) $$\{\begin{array}{c} \Delta \theta =(1-\frac{\sqrt{{\left(am_{11}+m_{14}\right)}^{2}+{\left(am_{21}+m_{24}\right)}^{2}}}{R_{2}})\cdot \theta \\ \Delta \theta_{S}=\frac{32T}{G\pi d^{4}}\cdot (\sqrt{R_{2}\tan\varphi }-\frac{m_{14}}{m_{11}}-L_{47}) \end{array},\;\mathrm{while}\;\varphi \neq 0$$

where θ is the measurement value of the angle encoder, T is the loading torque, and G is the shear modulus.

According to the error compensation model, the error $\Delta \theta_{S}$ caused by the change of the measurement area is a linear change value of the torque T, which can be compensated by subtracting the value of this part from the measured value of the sensor. The measurement error of the angle sensor can be divided into two cases: When φ=0, which is judged according to Formula (14), the error Δθ can be obtained according to Formula (2), so that the eccentric error of the actual measurement point in the X and Y directions can be calculated by the above model. When φ≠0, the coefficient of error Δθ with respect to θ can be obtained by substituting the manufacturing error of each part into the above model.

## 4. Simulation and Experiment

We analyzed the influence of geometric parameters on the measurement accuracy of an angle sensor based on the above model and used a self-collimator for validation experiments. Finally, a new angle measurement error compensation strategy was used to compare the test results of the TCMS system.

### 4.1. Simulation Verification

**Figure 5:**
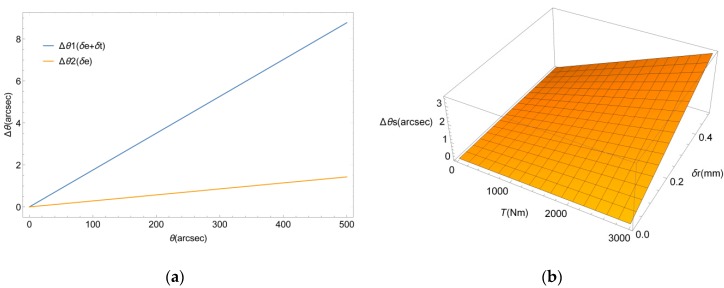
(**a**) Comparison of the influence of the tilt errors and eccentricity errors. (**b**) Influence of the relative position error.

The numerical analysis software MATLAB was used to simulate the proposed error compensation model. In order to analyze the influences of the tilt error and the relative position errors on the measurement results, the effects of the relative position error along the *x*-axis, the rotation error around the *y*-axis, and the translation error along the *y*-axis on the angle measurement error were taken as an example. The structure parameters of the measurement unit are shown in Table 2, and according to the machining accuracy of the components, the assemble errors δr, δe, and δt were set to 0.1 mm, 0.02 mm, and 0.02 rad, respectively. The simulation results of the measurement error are shown in Figure 5. The following conclusions can be drawn from the graph: (1) The measurement error was linear with the geometric errors as the measurement angle increased from 0 to 500 arcsec. (2) The measurement error increased after considering the tilt errors and eccentricity errors simultaneously. (3) The influence of the relative position error was less than that of the tilt error, so that $\Delta \theta_{S}$ increased by 3 arcsec as the loading torque increased from 0 to 3000 Nm.

### 4.2. Calibration Experiment

**Figure 6:**
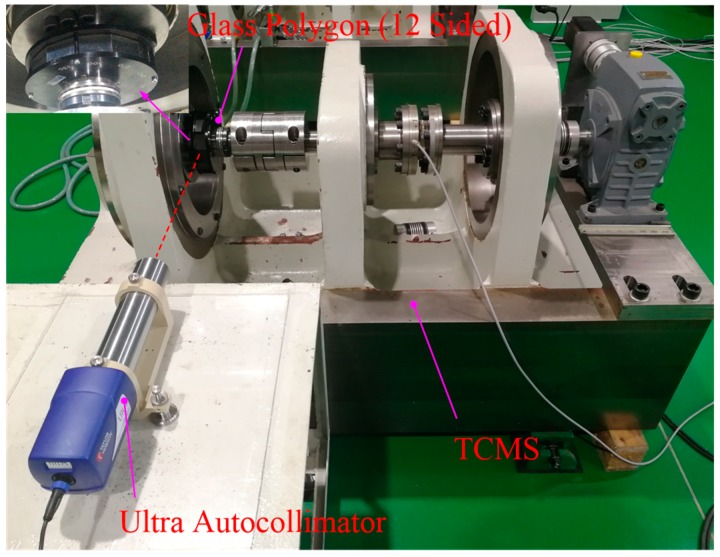
Verification experiment using an ultra-autocollimator.

To validate the error compensation method, a calibration experiment was performed on the torsional characteristics measurement system, which is illustrated in Figure 6. In particular, an ultra-autocollimator with a 12-sided glass polygon (Taylor Hobson; the measuring range was 1800 arcsec, the resolution was 0.001 arcsec, and the accuracy over the total range was 0.2 arcsec) was used to measure the torsion angle when the loading actuator was loaded with the given value. The experiment procedure was as follows:

1. Verify the geometric errors of each part of the measurement unit as shown in Table 3 and obtain the error compensation function based on the above model.
2. Select the appropriate output shaft that can simultaneously satisfy the mounting of the angle sensor and the glass polygon, so that the theoretical measurement points are consistent. Then, determine the theoretical measurement point.
3. A torsion angle is measured every 50 Nm in a load torque range of 1700 Nm.
4. Repeat (3) three times and take an average to obtain 34 angle values.
5. Repeat (2)–(4) using a circular grating angle encoder.
6. Compensate the measured values of the angle encoder according to the proposed error model, and compare the data before and after the compensation and the autocollimator.

**Figure 7:**
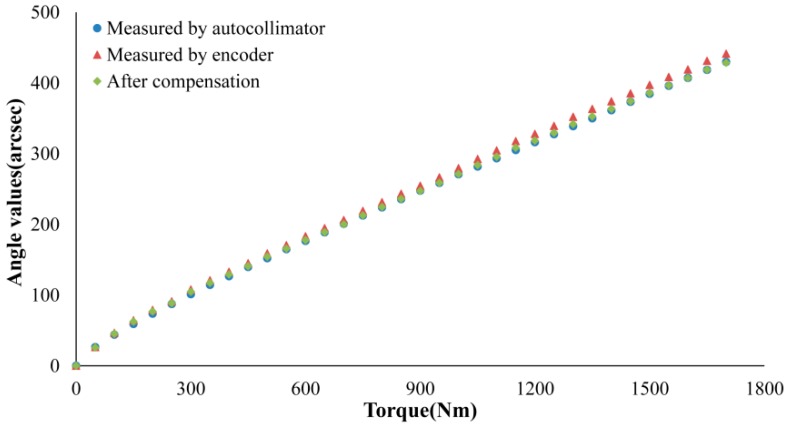
The chart of data comparison.

Figure 7 shows the experiment data. It can be seen that the compensated angle value was quite consistent with the amplitude of each point measured by the self-collimator, and the maximum of the angular error was reduced from 10.9 to 2.1 arcsec within the range measured after compensation. In addition, the angular measurement error reduced from 3.7% to 0.7%, as shown in Table 4. 

**Figure 8:**
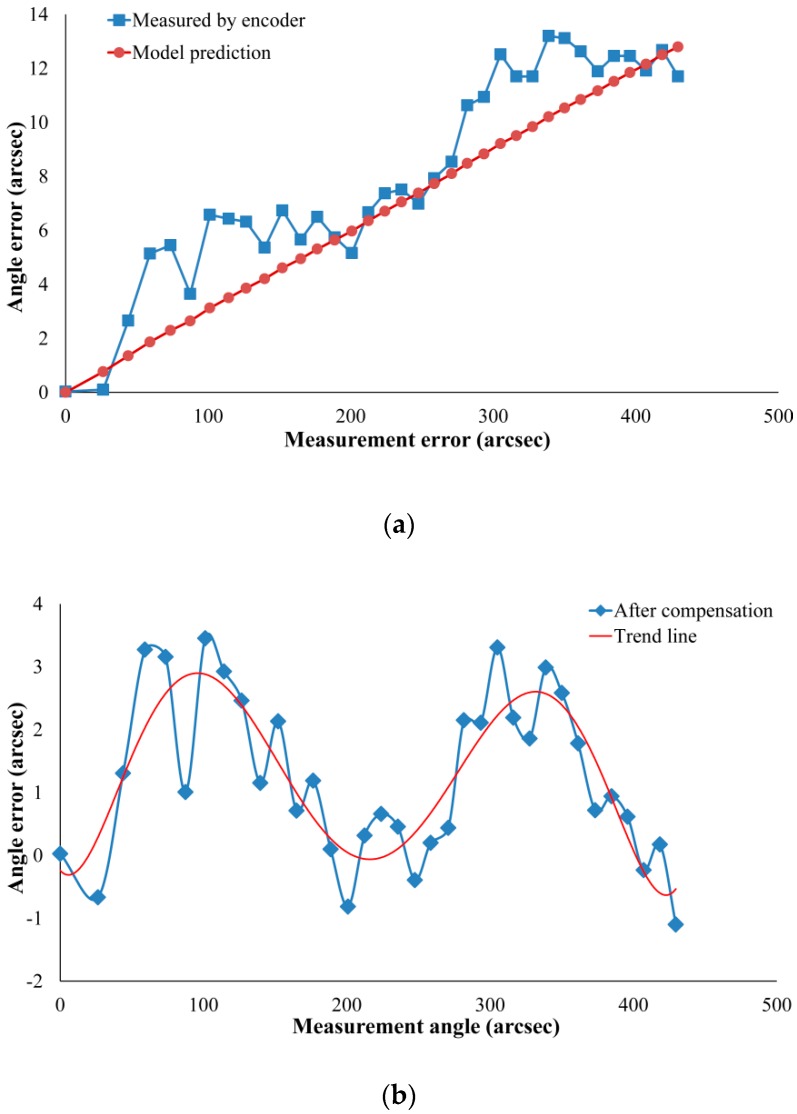
(**a**) Comparison between model prediction error and encoder measurement error. (**b**) Errors after compensation.

Figure 8a shows the difference between the actual error value and the model compensation value. The square dotted line indicates the actual error value, which is the difference of the measured values between the angle encoder and the autocollimator. The round dotted line indicates the compensation value of the error compensation model. It can be observed that the square and dotted lines had the same tendency when the angle increased, which indicates that the error compensation model was correct. In addition, the square line is slightly above the round dotted line and has significant sinusoidal periodic fluctuations, indicating that the actual error contained other error terms. In the actual situation, the compensation coefficient was a constant when the manufacturing error was determined. It can be seen from Figure 8a that the predicted error based on the model was linear. According to the error model, the measurement error of the angle sensor can be effectively reduced by analyzing the influence of manufacturing errors on the compensation coefficient and controlling the machining accuracy of the critical dimension of the components.

It can be seen from Figure 8b that the actual error value fluctuated within 2 arcsec, which was consistent with the system accuracy of the angle encoder. For the case where the actual error value was larger than the compensation value by 1–2 arcsec, it can be considered as the following two cases: (a) The geometric error is averaged along the error-sensitive direction in the error model. However, the geometric error is in a fixed direction in the actual case, so the compensation value deviates from the actual error value. (b) Only the main geometric errors are considered in this paper, while the deformation of the measurement point is not taken into consideration in the measurement process.

Consequently, the above experimental results and discussions show that the proposed error model and compensation method can effectively predict the angular measurement error caused by geometric errors. In addition, the error compensation model is a function of the part manufacturing error and the sensor angle. Therefore, the compensation coefficient can be quickly solved using MATLAB software. According to the LabVIEW programming language of the measurement system, the real-time compensation of angle measurements can be achieved by writing compensation coefficients for the measurement program.

### 4.3. Measurement of Torsional Characteristics for RV Reducer

**Figure 9:**
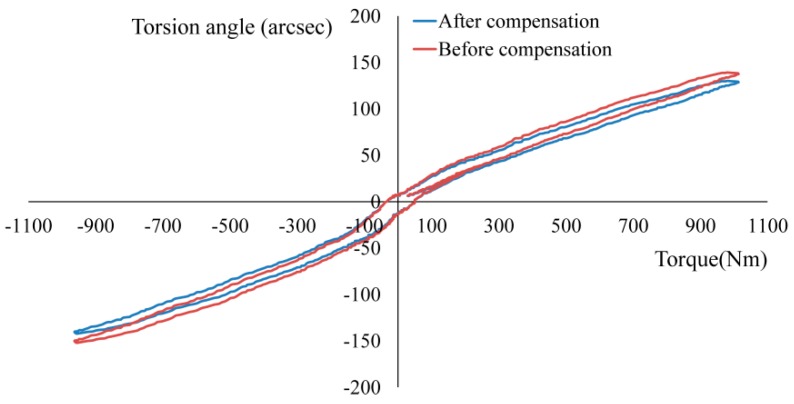
The measurement hysteresis curve of RV-100C.

Three types of reducers (Nabtesco’s RV-110E, RV-60N, and RV-100C) were used in this study to validate the accuracy of the measurement and the reasonableness of the error compensation. Figure 9 shows the measurement results of the hysteresis curve for the RV reducers. The backlash and torsional stiffness were evaluated and compared with the sample data generated by the sample calculation method. The comparison results are shown in Table 5. 

It can be observed from Figure 9 that the measured hysteresis curve is smooth and closed, indicating that the angle measured by the sensor was consistent with the actual torsion angle. On the other hand, the values of backlash were within the sample range and were consistent with the actual situation according to Table 5. The difference between the measured value after compensation and the sample value of the torsional stiffness was 3.13, 2.51, and 2.79 Nm/arcmin, respectively. The system measurement errors decreased by 8.3%, 9.2%, and 13.4% before and after angular error compensation, respectively. So, the measurement results have also validated the proposed error compensation method. 

## 5. Conclusions

In this paper, the influence of manufacturing and assembly errors on the measurement accuracy of an angle encoder was analyzed from the perspective of measurement structure. Based on the traditional error compensation model with calibration data, an angular sensor measurement model considering geometric errors was proposed. The process of error action was considered to be the motion between rigid bodies according to the characteristics of the new model. The geometric error was decomposed by the error-sensitive direction, and the error transfer matrix was obtained based on the multibody system theory and the DH transformation principle. Therefore, the relationship between the spatial position parameters of the measurement point and the geometric errors could be established. Next, the mathematical function of the measurement error was derived, with the established error compensation strategy. The simulation results showed that these errors, including translation errors along the *x*-axis, eccentricity errors, and tilt errors along the mounting surface, have an obvious influence on the angle measurement error. Further, the rationality of the model for error prediction was also validated. In addition, the results of the autocollimator in the comparison verification experiment proved the correctness of the proposed error compensation method. The measurement error of the encoder after compensation was reduced from 3.7% to 0.7% compared to the measurement data of the autocollimator. The measurement error of TCMS decreased by 8.3% to 13.4% before and after angular error compensation on three types of RV reducer, and the measurement results also validated the proposed error compensation method. 

However, the compensation model is still insufficient because it does not compensate for the error caused by bending deformation during the measurement. In fact, we observed changes in the measurement points caused by deformation during the comparison experiment, which also caused angle measurement error. Therefore, the compensation model still needs to be improved, although its performance is good. We will improve the reliability of the measurement system in the future.

## Figures and Tables

**Table 1:** Transfer errors of the adjacent components.

Adjacent Parts	$\Delta x_{ij}$	$\Delta y_{ij}$	$\Delta z_{ij}$	$\Delta \alpha_{ij}$	$\Delta \beta_{ij}$	$\Delta \gamma_{ij}$
*S1-S2*	-	$\delta e_{12}$	$\delta e_{12}$	-	$\delta t_{12}$	$\delta t_{12}$
*S2-S3*	$\delta r_{23}$	$\delta e_{23}$	$\delta e_{23}$	-	$\delta t_{23}$	$\delta t_{23}$
*S3-S4*	$\delta r_{34}$	$\delta e_{34}$	$\delta e_{34}$	-	$\delta t_{34}$	$\delta t_{34}$
*S1-S5*	$\delta r_{15}$	$\delta e_{15}$	$\delta e_{15}$	-	$\delta t_{15}$	$\delta t_{15}$
*S5-S6*	-	$\delta e_{56}$	$\delta e_{56}$	-	$\delta t_{56}$	$\delta t_{56}$

**Table 2:** Structure parameters of the measurement unit.

Parameters	*L_23_*	*L_34_*	*L_15_*	*L_47_*	*R_2_*	*d*
Values	5 mm	67 mm	120 mm	38 mm	35 mm	58 mm

**Table 3:** Inspection values of the parts.

Component	Δl (mm)	Δd (mm)	Δτ (rad)	Δc (mm)
1	-	0.084	0.005	-
2	0.016	−0.026	0.012	0.022
3	−0.017	0.005	0.005	-
4	0.045	0.075	0.018	-
5	−0.025	−0.042	0.008	-
6	-	0.002	-	-

**Table 4:** Measurement data and compensation data.

Torque (Nm)	Measured by Autocollimator (arcsec)	Measured by Angle Encoder (arcsec)	After Compensation (arcsec)
0	0.07	0.08	0.08
50	26.36	26.46	25.67
100	43.97	46.63	45.31
150	59.22	64.36	62.58
200	73.59	79.04	76.83
250	87.45	91.1	88.48
300	101.23	107.81	104.77
350	114.42	120.85	117.42
400	126.67	132.99	129.19
450	139.72	145.08	140.89
500	152.13	158.87	154.31
550	164.97	170.63	165.68
600	176.61	183.11	177.81
650	188.77	194.51	188.85
700	200.86	206.02	199.99
750	212.45	219.12	212.75
800	223.97	231.34	224.62
850	235.72	243.23	236.16
900	247.57	254.56	247.13
950	258.62	266.55	258.79
1000	270.86	279.4	271.27
1050	281.77	292.4	283.95
1100	293.54	304.48	295.67
1150	305.12	317.64	308.43
1200	316.2	327.95	318.39
1250	327.64	339.34	329.49
1300	338.9	352.14	341.89
1350	350.12	363.24	352.71
1400	361.42	374.05	363.20
1450	373.33	385.22	374.05
1500	384.75	397.21	385.69
1550	395.98	408.44	396.6
1600	407.21	419.13	406.98
1650	418.54	431.22	418.71
1700	429.62	441.32	428.52

**Table 5:** Comparison of measurement results with sample data.

Reducer	Backlash (arcsec)		Torsional Stiffness (Nm/arcmin)			
	Measured	Sample	Measured	After Compensation	Sample	Rate
RV-60N	31.49	≤60	187.49	203.13	200	8.3%
RV-100C	18.95	≤60	469.25	512.79	510	9.2%
RV-110E	25.15	≤60	260.71	295.67	294	13.4%
